# cryoEM structure of NINJ1, a small cytotoxic membrane protein.

**DOI:** 10.1063/4.0000868

**Published:** 2025-10-27

**Authors:** Ishan Deshpande

**Affiliations:** Genentech, South San Francisco, CA, USA

## Abstract

Lytic cell death culminates in plasma membrane rupture, which releases large intracellular molecules to augment the inflammatory response. Plasma membrane rupture is mediated by the effector membrane protein NINJ1, which polymerizes and ruptures the membrane via its hydrophilic face. How NINJ1 is restrained under steady-state conditions to ensure cell survival remains unknown. Here we describe the molecular underpinnings of NINJ1 inhibition. Using cryoEM, we determined the structure of inactive-state NINJ1 bound to the newly developed nanobody Nb538. Our structure-function studies demonstrate how NINJ1 is kept inactive in its basal state to prevent unprovoked plasma membrane rupture and cell death.

